# Genome-wide DNA methylation and RNA-seq analyses identify genes and pathways associated with doxorubicin resistance in a canine diffuse large B-cell lymphoma cell line

**DOI:** 10.1371/journal.pone.0250013

**Published:** 2021-05-07

**Authors:** Chia-Hsin Hsu, Hirotaka Tomiyasu, Chi-Hsun Liao, Chen-Si Lin

**Affiliations:** 1 Department of Veterinary Medicine, School of Veterinary Medicine, National Taiwan University, Taipei, Taiwan; 2 Department of Veterinary Internal Medicine, Graduate School of Agricultural and Life Sciences, The University of Tokyo, Tokyo, Japan; University of Southern California, UNITED STATES

## Abstract

Doxorubicin resistance is a major challenge in the successful treatment of canine diffuse large B-cell lymphoma (cDLBCL). In the present study, MethylCap-seq and RNA-seq were performed to characterize the genome-wide DNA methylation and differential gene expression patterns respectively in CLBL-1 8.0, a doxorubicin-resistant cDLBCL cell line, and in CLBL-1 as control, to investigate the underlying mechanisms of doxorubicin resistance in cDLBCL. A total of 20289 hypermethylated differentially methylated regions (DMRs) were detected. Among these, 1339 hypermethylated DMRs were in promoter regions, of which 24 genes showed an inverse correlation between methylation and gene expression. These 24 genes were involved in cell migration, according to gene ontology (GO) analysis. Also, 12855 hypermethylated DMRs were in gene-body regions. Among these, 353 genes showed a positive correlation between methylation and gene expression. Functional analysis of these 353 genes highlighted that TGF-β and lysosome-mediated signal pathways are significantly associated with the drug resistance of CLBL-1. The tumorigenic role of TGF-β signaling pathway in CLBL-1 8.0 was further validated by treating the cells with a TGF-β inhibitor(s) to show the increased chemo-sensitivity and intracellular doxorubicin accumulation, as well as decreased p-glycoprotein expression. In summary, the present study performed an integrative analysis of DNA methylation and gene expression in CLBL-1 8.0 and CLBL-1. The candidate genes and pathways identified in this study hold potential promise for overcoming doxorubicin resistance in cDLBCL.

## Introduction

Canine diffuse large B-cell lymphoma (cDLBCL) is one of the most frequently diagnosed malignancies in dogs. It resembles human lymphoma in many essential ways, such as characteristic translocations and molecular abnormalities, as well as similar therapeutic responses to chemotherapy [1]. Multidrug chemotherapy that is doxorubicin-based remains the treatment of choice and extends the life span of dogs affected by cDLBCL; however, cDLBCL remains incurable, with variable survival time and high relapse rates due to drug resistance [1, 2].

Since drug resistance is the major limiting factor in the successful treatment of cDLBCL with doxorubicin-based chemotherapy, understanding the mechanisms causing drug resistance will significantly improve therapeutic strategies for fighting cDLBCL and hence increase the survival rates. Multiple mechanisms contribute to drug resistance, including increased drug efflux, changes in drug metabolism or drug target, and activation of downstream or parallel signal transduction pathways [3, 4]. Increasing evidence has shown that genetic mutations play critical roles in drug resistance; however, the relatively rapid appearance or the reversibility of non-responsiveness to drug treatment cannot be explained by genetics [4]. Also, research has revealed that aberrant DNA methylation may occur at a much higher frequency than gene mutation and therefore have a greater impact on the acquisition of resistance to anticancer drugs [5]. Thus, it is critical to understand the DNA methylation profile of resistant cDLBCL to achieve a breakthrough in cancer treatment.

Aberrant DNA methylation has gained increasing attention in the field of cancer. Promoter DNA hypermethylation, which induces gene silencing of tumor suppressor genes, has been generally recognized as one of the critical features of cancer [6]. On the other hand, recent studies have shown that gene body methylation is positively correlated with gene expression [7–9]. Since methylation in both promoter and gene-body regions regulates gene expression, the details of the genome-wide methylation profile of doxorubicin-resistant cDLBCL should be further investigated. The strategy we used here to study the methylation profile is called MethylCap, which is based on the capture of methylated DNA with the MBD domain of methyl-CpG binding protein 2 (MeCP2) [10]. With this strategy, many novel regions that are hypermethylated in biological samples can be revealed [11].

To date, no reports on genome-wide DNA methylation and transcriptome analysis of resistant cDLBCL have been published. CLBL-1, a cDLBCL cell line, has been established from clinical specimens as a powerful research tool for comparative tumor biology and cancer drug development [12]. We previously developed a doxorubicin-resistant CLBL-1, named CLBL-1 8.0, which allowed us to study the doxorubicin resistance of cDLBCL [13]. In the present study, with CLBL-1 8.0 and CLBL-1 as control, we intend to investigate the role of epigenetically dysregulated genes and pathways with a potential role in the drug resistance of cDLBCL.

## Materials and methods

### Cell lines

CLBL-1 was kindly provided by Dr. Barbara C. Rütgen’s laboratory (University of Veterinary Medicine Vienna, Vienna, Australia). The cells were grown in RPMI medium at 37°C with 10% fetal bovine serum (FBS; Caisson, USA) in a humidified atmosphere containing 5% CO2. CLBL-1 8.0, a doxorubicin-resistant CLBL-1 established in our previous study (Chen et al., 2019), was maintained in RPMI containing 10% FBS and 8 μM of doxorubicin hydrochloride (Merck, Germany).

### MethylCap sequencing

Genomic DNA prepared from CLBL-1 and CLBL-1 8.0 was extracted using the Genomic DNA isolation Kit (GeneDirex, Taiwan) and fragmented by ultrasound-mediated DNA shearing (M220 Focused-ultrasonicator^TM^, Covaris, USA) set to obtain fragments with an average length of 400 bp. We employed the MethylCap kit (Diagenode, USA) to obtain methylated DNA fragments and followed by the library preparation for the captured DNA using the QIAseq Ultralow Input Library Kit (Qiagen, Germany). The library was quantitatively measured by Kapa Library Quantification Kit for the Illumina platform (Kapa Biosystems, USA) and Qubit dsDNA HS Assay kit (ThermoFisher, USA). The library was sequenced using a 150 bp paired-end protocol on an Illumina Novaseq (Illumina, USA). The data from MethylCap sequencing in this study are available in the NCBI GEO database with the accession number GSE149925 (https://www.ncbi.nlm.nih.gov/geo/query/acc.cgi?acc=GSE149925).

### RNA sequencing

RNA sequencing libraries were prepared with 3 μg of total RNA using the NEBNext® Ultra™ RNA Library Prep Kit for Illumina® (NEB, USA), and index codes were added to attribute sequences to each sample. RNA sequencing was performed in duplicate using Illumina Novaseq 6000. The data from RNA-seq are available in the NCBI GEO database with the accession number GSE149384 (https://www.ncbi.nlm.nih.gov/geo/query/acc.cgi?acc=GSE149384). The details of data processing for Methylcap-seq and RNA-seq were described in *S1 File*.

### Data processing of Methylcap-seq and RNA-seq

For Methlycap-seq, the sequencing quality processing tool (FASTX-Toolkit http://hannonlab.cshl.edu/fastx_toolkit/) was used to filter out low quality sequencing reads. The command was “fastq_quality_filter–Q33 –q 20 –p 70”, where “-q 20” indicated that the minimum phred score was 20 (20 means the sequencing error rate is 1% to a base) and “-p 70” indicated that the minimum percent of bases must have “-q” quality ≥ 70%. Sequencing reads were retained if both forward and reverse sequencing reads passed the previous step. BWA (http://bio-bwa.sourceforge.net/) was used to align sequencing reads with dog genome sequences (CanFam3.1) obtained from NCBI. Then MACS2 (https://github.com/taoliu/MACS) was employed to identify hypermethylated peaks according to the results of sequencing alignment. The thresholds of hypermethylated peaks were fold-enrichment > 4 and p-value < 0.01. The gene information downloaded from NCBI and the genomic location of enhancer downloaded from https://github.com/lingchen42/EnhancerCodeConservation were used to annotate hypermethylated peaks.

For RNA-seq, the sequencing quality processing tool (FASTX-Toolkit http://hannonlab.cshl.edu/fastx_toolkit/) was used to filter out low quality sequencing reads. Sequencing reads were retained if both forward and reverse sequencing reads passed the previous step. HISAT2 (http://daehwankimlab.github.io/hisat2/) was used to align sequencing reads with dog genome sequences (CanFam3.1) obtained from NCBI. Then StringTie (https://ccb.jhu.edu/software/stringtie/) was employed to evaluate the expression levels of transcripts. The criteria of transcripts per million (TPM) > 5 in one sample and log2 fold-change of ≥ 0.67 or ≤-0.67 were applied to identify differentially expressed genes. For gene function analysis, the genes hypermethylated in the gene body (methylation fold change ≥3 (CLBL-1 8.0/CLBL-1)) and up-regulated mRNA expression (log2 fold-change ≥1.5) were applied to do gene ontology (GO) and pathway enrichment analyses with David Bioinformatics Resources 6.8 (https://david.ncifcrf.gov/). The false discovery rate (FDR) for GO and KEGG analysis in DAVID database is calculated by the adaptive linear step-up adjusted p-values and the lowest slope method to estimate the number of true NULL hypotheses. Because the reference lists of GO terms and pathway might have a slight difference between distinct database leading to the different FDR. The gene candidates from each study criterion were also applied into the PANTHER platform for GO enrichment analysis, which service provided in the Gene Ontology website, to confirmed the enrichment results in this study. As same as DAVID, the FDR in PANTHER is also calculated by Benjamini-Hochberg method. The significantly enriched pathways were defined by nominal P<0.01 and FDR<0.25.

To understand the interactions between different genes, we used the Search Tool for the Retrieval of Interacting Genes (STRING) to construct the PPI network. The interaction score ≥ 0.4 was selected as significant and retained. The result of STRING analysis was further analyzed by Cytoscape v.3.7.1 [14, 15]. In Cytoscape, we used Molecular Complex Detection (MCODE) [16] to screen the important module in the PPI network, with degree cutoff = 2, node score cutoff = 0.2, k-core = 2, and max depth = 100, and the top 5 hub genes of the PPI network were analyzed by CytoHubba plug-in [17].

### Methylation-specific PCR

500 ng of DNA extracted from CLBL-1 and CLBL-1 8.0 was bisulfite-treated with the EZ DNA Methylation-Gold kit (Zymo Research, USA). ZymoTaq Premix was used for Methylation-Specific PCR (MSP) and according to the primers designed by MethPrimer 2.0 (http://www.urogene.org/methprimer2/) (S1 Table in S1 File). These selected genes and primer sequences were described in S1 Table in S1 File. Amplification was performed using AmpliTaq Gold DNA polymerase (Applied Biosystems, USA). The PCR reaction conditions included a pre-denaturation (95°C for 2 min), followed by 40 cycles of denaturation (95°C for 40 sec), annealing (55°C for 40 sec), and extension (72°C for 1 min), and then a final long extension (72°C for 8 min). The PCR products were electrophoresed through a 3% agarose gel and stained with ethidium bromide for visualization.

### Quantitative RT-PCR

The amounts of the selected hypermethylated/upregulated genes extracted from the MethylCap-seq and RNA-seq data were validated by RT-qPCR. The candidate mRNAs chosen for validation were listed in S2 Table in S1 File. GAPDH and OAZ-1 were used as the control gene [18]. 2 μg of total RNA was reverse transcribed using Deoxy^+^ HiSpec reverse transcriptase (Yeastern, Taiwan), and qPCR was performed by SensiFAST SYBR Lo-ROX Kit (Bioline, Germany) in AriaMx Real-time PCR System (Agilent, USA). PCR amplification were accordingly done: initial denaturation at 95°C for 2 min, followed by 40 cycles with denaturation at 95°C for 30 sec, annealing at 60°C for 30 sec, and extension at 72°C for 20 sec, followed by a final extension at 72°C for 7 min. The no-template control and a minus-RT control were included in each PCR reaction. The melting curve analysis showed no mis-priming in any of the reactions. Fold changes of gene expression levels were calculated by using the 2^(-ΔΔCT)^ method. The validation tests were independently repeated at least three times, and the results are displayed as mean ± SEM. Student’s *t*-test (two-tailed) was used to determine the statistical significance of differences between CLBL-1 and CLBL-1 8.0. *P* < 0.05 was considered statistically significant.

### Cytotoxicity assay

Cytotoxicity was evaluated with WST-1 according to the instruction manual (Roche, Germany). Approximately 4×10^4^ CLBL-1 and CLBL-1 8.0 cells were seeded in 96-well plates with the indicated concentrations of a TGF-β receptor inhibitor, SB505124 (Merck Sigma-Aldrich), treatment 24 hours prior to doxorubicin (Dox) treatment. After 48 hours of Dox treatment, WST-1 was added to each well, and the plates were incubated for 3 hours, followed by absorbance measurement at 450 nm by SpectraMax M5 microplate reader (Molecular Devices, USA).

### Cellular uptake of doxorubicin

CLBL-1 and CLBL-1 8.0 cells were preincubated in energy-supplied buffer (PBS plus 10% FBS with 10 mM glucose) for 1 hour at 37°C, and then with 10 μM doxorubicin in energy-supplied buffer with or without SB505124 for 2 hours at 37°C. After washing with ice-cold PBS three times, the cells were resuspended with 1% Triton X-100 and 0.2% SDS in 20 mM phosphate buffer (pH 7.4). Finally, the fluorescence was determined using flow cytometry. Flow cytometry was performed on a FACS LSRFortessa^TM^ flow cytometer (Becton Dickinson, USA). Data from 1×10^4^ cells were collected and analyzed using the FACSDiva software (Becton Dickinson).

### Western blot analysis

Cell lysates of CLBL-1 and CLBL-1 8.0 were treated with SB505124 (5 μM) for 24 hours and harvested for immunoblotting. The protein lysates (30 μg) were subjected to SDS-PAGE and blotted from 10% (w/v) polyacrylamide gel to a hydrophobic polyvinylidene difluoride (PVDF) membrane. After the 2-hour blocking of the PVDF membrane in TBS, 0.05% Tween 20 (TBST) plus 5% skim milk, the following primary antibodies diluted in TBST was used: ABCB1/P-gp (1:50) (ThermoFisher), TGF-β receptor II (1:1000), Phospho-Smad2/3 (1:500), Smad2 (1:1000), β-actin loading control monoclonal antibody (Cell Signaling Technology, USA) for 2h. Then these membranes were washed in TBST and then incubated for 1 h at room temperature with horseradish peroxidase conjugated anti-mouse or rabbit IgG for 1 hour. Finally, the membrane was washed with TBST and developed with a chemiluminescent peroxidase substrate (Merck Sigma-Aldrich) and pictured using the Geliance 600 Imaging System (PerkinElmer, USA).

## Results

### Differential DNA methylation in CLBL-1 8.0

**Fig 1:**
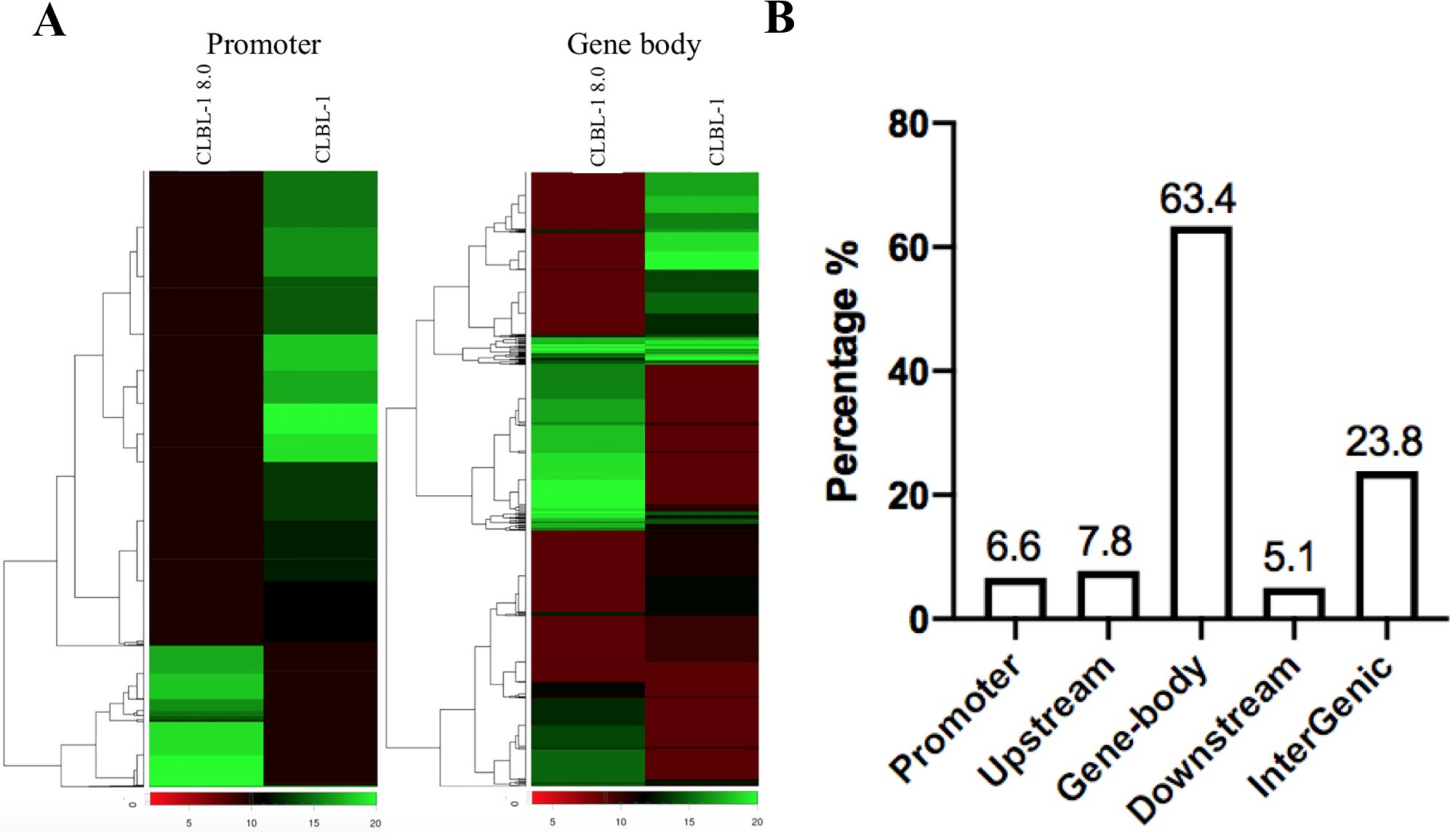
Genomic distribution of methylation changes in CLBL-1 and CLBL-1 8.0. (A) Heatmap depicting DNA methylation pattern between CLBL-1 and CLBL-1 8.0 in the promoter and gene-body regions. A greener color indicates the larger value of peak fold enrichment. (B) Bar chart showing the genomic distribution of CpG sites with altered DNA methylation patterns in CLBL-1 8.0 as compared to CLBL-1. “Upstream” is defined as 5000 bp upstream of Transcription start site (TSS), and “Promoter” is defined as 3000 bp upstream of TSS.

To investigate the role of DNA methylation in doxorubicin-resistant cDLBCL, DNA methylation analysis of CLBL-1 8.0 using MethylCap was performed and compared with CLBL-1 as control. Sharp differences in the methylation patterns of both promoter and gene-body regions were detected between CLBL-1 8.0 and CLBL-1, as shown in Fig 1A. Methylated peaks with fold-change greater than 4 and a P value of less than 0.01 were defined as differentially methylated regions (DMRs); totals of 20289 hypermethylated and 38362 hypomethylated DMRs were detected. Among these, 1339 hypermethylated and 4861 hypomethylated DMRs were in promoter regions, while 12855 hypermethylated and 18904 hypomethylated DMRs were in gene-body areas. Fig 1B shows the distribution and location of the hypermethylated DMRs; 6.6% of the hypermethylated DMRs were located in the promoter regions. A high percentage (63.4%) of hypermethylated DMRs were distributed within the gene body, and 7.8%, 5.1% and 23.8% of the hypermethylated DMRs were found to be clustered in upstream, downstream, and intergenic regions, respectively (Fig 1B).

### Differential gene expression in CLBL-1 8.0

To analyze the biological relevance of methylation changes, the gene expressions from both CLBL-1 and CLBL-1 8.0 were examined. RNA samples from both cell lines were of excellent quality, confirmed by RIN values of 9.90 for CLBL-1 and 10.00 for CLBL-1 8.0. In total, we obtained 24.8 M and 23.7 M sequence reads for CLBL-1 and CLBL-1 8.0, respectively. After filtering out low-quality bases and reads from the datasets before read mapping, 19.8 M reads for CLBL-1 and 19.1 M reads for CLBL-1 8.0 were mapped to the canine genome. The criteria of transcripts per million (TPM) >5 in one sample and log2 fold-change ≥ 0.67 or ≤ -0.67 were applied to identify differentially expressed genes. Upregulated and downregulated genes numbered were 1127 and 602, respectively. The different gene expression patterns between CLBL-1 8.0 and CLBL-1 was shown in S1 Fig in S1 File. Functional analyses including GO terms and KEGG pathways for differentially expressed genes (DEGs) were performed (S3 and S4 Tables in S1 File). The enriched KEGG pathways of these DEGs revealed “endocytosis”, “lysosome”, “apoptosis”, “Ras signaling pathway”, and a variety of cancer-associated processes. These results correlate with our previous findings in CLBL-1 8.0 which possesses aggressive drug resistance to doxorubicin.^13^ It has been demonstrated that chemoresistance could partially result from the trap and sequestration of anticancer drugs within lysosomes [19].

### Potential pathways and specific genes related to doxorubicin resistance were revealed by analyzing hypermethylated/upregulated genes in gene-body regions in CLBL-1 8.0

**Fig 2:**
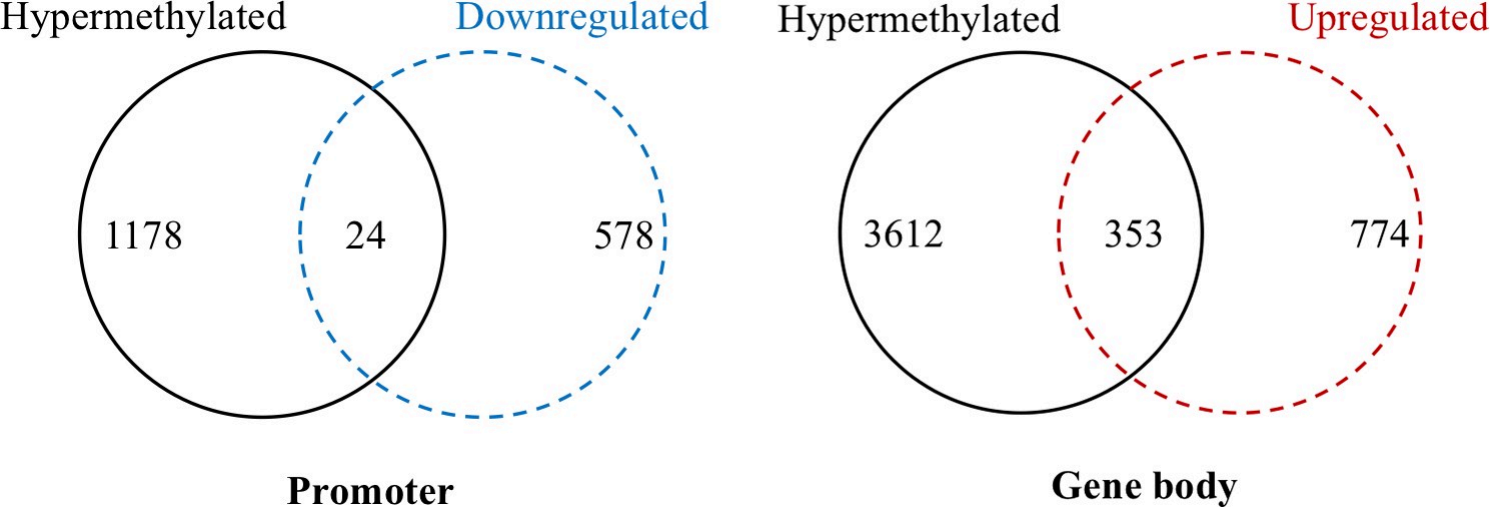
Integrative analysis of genes with differential hypermethylation and differential expression in the promoter and gene-body regions in CLBL-1 8.0. (A) The total number of hypermethylated/downregulated genes in promoter regions. A total of 24 genes were hypermethylated and downregulated in expression. (B) The total number of hypermethylated/upregulated genes in the gene-body areas. A total of 353 genes were hypermethylated and upregulated in expression.

**Fig 3:**
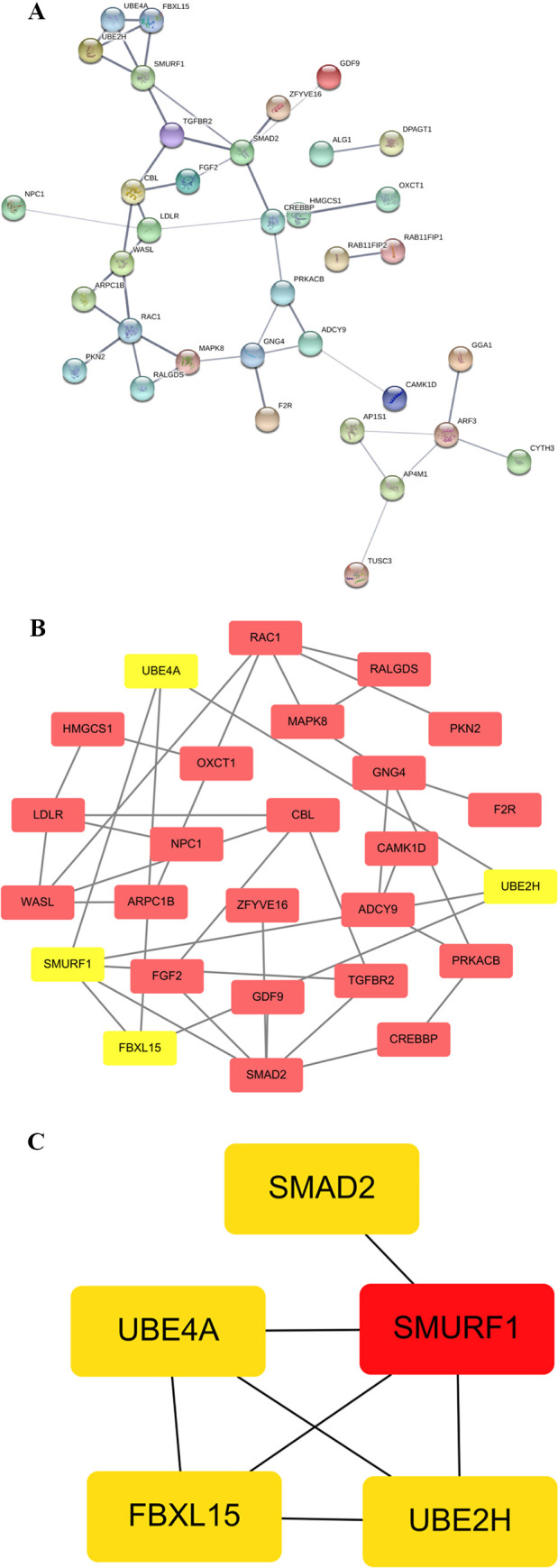
PPI network and analysis of hub genes of hypermethylated/upregulated genes in gene-body regions belonging to the enriched KEGG pathways in CLBL-1 8.0. (A) PPI network: the thickness of network edges correlates with the confidential score provided by the STRING database. A thicker edge indicates a higher confidence score of the interaction. (B) Clustering analysis of the PPI network. The most important modules and genes obtained using MCODE in Cytoscape. (C) Hub genes identified by cytoHubba tool kits in Cytoscape. The selected nodes are shown with a color scheme from highly essential (red) to crucial (yellow).

It is well known that promoter hypermethylation in cancer leads to the silencing of downstream genes [20]. To determine whether promoter hypermethylation could silence gene expression in doxorubicin-resistant cDLBCL, we did an integrative analysis of the 1202 genes and found hypermethylation in the promoter regions with the 602 differentially expressed genes (DEGs) in CLBL-1 8.0. Among this, promoters of 24 downregulated DEGs were hypermethylated (Fig 2A). In addition to promoter hypermethylation, which induces gene silencing, hypermethylation in gene-body regions has been reported to be positively correlated with gene expression [7–9]. Thus, the same procedure was conducted for the integrative analysis of the 3965 genes showing hypermethylation in the gene-body regions with the 1127 DEGs in CLBL-1 8.0. The gene bodies of 353 upregulated DEGs were hypermethylated (Fig 2B), suggesting that gene body hypermethylation might have functional consequences. GO analysis of these 353 genes identified 2 Biological Process (BP), 5 Cellular Component (CC) and 3 Molecular Function (MF) GO terms as significantly enriched (p < 0.01) (Table 1). Besides, a Kyoto Encyclopedia of Genes and Genomes (KEGG) pathway analysis revealed 5 significantly enriched KEGG pathways (p < 0.01) (Table 2). Most of these GO and KEGG pathways were related to cancer, including “TGF-β signaling pathway”, “SMAD binding”, “protein serine/threonine kinase activity”, “ubiquitin-like protein transferase activity”, “Golgi to lysosome transport”, “Lysosome”, “pancreatic cancer”, “Ras pathway”, “ubiquitin mediated proteolysis”, and “protein modification by small protein conjugation or removal”. These hypermethylated/upregulated genes in gene-body regions belonging to the enriched KEGG pathway (p < 0.01) (Table 2) were mapped to the protein–protein interaction (PPI) annotations from the STRING database [21, 22], as shown in Fig 3A. In this PPI network, the most crucial module was obtained using Cytoscape (MCODE plug-in) (Fig 3B) [14–16]. The top 5 genes ranked in the top 10% of connectivity in candidate modules [23], including *SMURF1*, *UBE4A*, *UBE2H*, *FBXL15* and *SMAD2*, were identified as hub genes by CytoHubba plug-in (Fig 3C) [17], and 4 of them were consistent with their enrichment in the top module analyzed by MCODE (showed in yellow in Fig 3B). By investigating the interactions among the hypermethylated/upregulated genes using PPI, Cytoscape, and CytoHubba plug-in, these hub genes were identified to reveal their potentially close involvement in doxorubicin resistance.

**Table 1:** Significantly enriched GO terms in hypermethylated/upregulated genes in gene-body regions in CLBL-1 8.0.

Category	GO ID	GO term	# of involved genes	p-value	FDR	involved gene list (gene names)
BP	GO:0090160	Golgi to lysosome transport	5	1.97E-06	4.62E-03	AP4M1,CLN3,CCDC91,SORT1
BP	GO:0070647	protein modification by small protein conjugation or removal	25	7.88E-05	4.62E-02	USP32,USP33,MAGEF1,DTX2,CBL,RNF157,RNF214,RNF216,HECTD2,ATXN7,RNF111,UBE2H,SHPRH,RNF24,SMURF2,SMURF1,NSMCE1,UBE4A,BFAR,USP28,PJA2,PIAS2,SPSB2,KCTD13,STX1A
CC	GO:0005225	volume-sensitive anion channel activity	5	1.33E-06	2.80E-03	TTYH3,LRRC8C,LRRC8D,LRRC8B,CLCN3
CC	GO:0019787	ubiquitin-like protein transferase activity	17	7.78E-05	4.69E-02	UBE2H,SHPRH,RNF24,SMURF2,SMURF1,NSMCE1,UBE4A,BFAR,DTX2,CBL,PJA2,PIAS2,RNF157,RNF214,HECTD2,KCTD13,RNF111
CC	GO:0004930	G protein-coupled receptor activity	3	9.19E-05	4.09E-02	F2R,C3AR1,CELSR2
CC	GO:0019899	enzyme binding	40	7.39E-05	5.29E-02	RB1,ERRFI1,BMPR2,MVP,USP33,SERPINE1,TULP3,NEDD9,CBL,NR3C1,RPAP2,RHOBTB1,GGA1,STRIP1,MAPK8,ZNHIT1,KAT8,RICTOR,RAC1,RAB11FIP2,PRKACB,WDTC1,SMAD2,PARP4,RFC2,IL1R1,RHBDD2,SORT1,EIF2AK3,BFAR,MAPK8IP3,PJA2,PIAS2,HINFP,BACE1,TFAP4,PKP2,POT1,PKN2,STX1A
CC	GO:0034212	peptide N-acetyltransferase activity	6	1.12E-04	4.73E-02	NAA60,CREBBP,ESCO1,LOC475708,KAT8,NAA16
MF	GO:0046332	SMAD binding	4	2.28E-05	1.61E-02	SMAD2,SMURF2,SMURF1,TGFBR2
MF	GO:0097367	carbohydrate derivative binding	16	3.20E-05	1.93E-02	ARF3,CAMK2D,UBE2H,CAMK1D,BMPR2,SPATA5,CTSS,RHOBTB1,TGFBR2,CLN3,MAPK8,PKN2,RAC1,PRKACB
MF	GO:0004674	protein serine/threonine kinase activity	7	5.02E-05	2.65E-02	CAMK2D,MAPK8,CAMK1D,BMPR2,PKN2,PRKACB,TGFBR2

**Table 2:** Significantly enriched KEGG pathways in hypermethylated/upregulated genes in gene body regions in CLBL-1 8.0.

KEGG ID	KEGG name	# of involved genes	p-value	FDR	involved gene list (gene names)
cfa04912	Gonadotropin-releasing hormone receptor pathway	7	3.89E-06	2.17E-04	SMAD2,MAPK8,CREBBP,BMPR2,PLA2G4A,RAC1
cfa04350	TGF-β signaling pathway	7	4.60E-05	7.69E-03	BMPR2, TGFBR2, ZFYVE16, CREBBP, SMURF1, SMAD2, SMURF2
cfa04014	Ras Pathway	8	6.70E-05	2.33E-03	MAPK8,RAC1,RALGDS, NMDAR, ERK, RAS, PI3K, TIAM1, LAT
cfa04142	Lysosome	8	3.52E-04	2.16E-02	SORT1, CTSS, GGA1, CLN3, NPC1, GUSB, AP4M1, AP1S1
cfa05212	Pancreatic cancer	6	4.52E-04	2.32E-02	RALGDS, MAPK8, TGFBR2, RAC1, SMAD2, RB1

**Fig 4:**
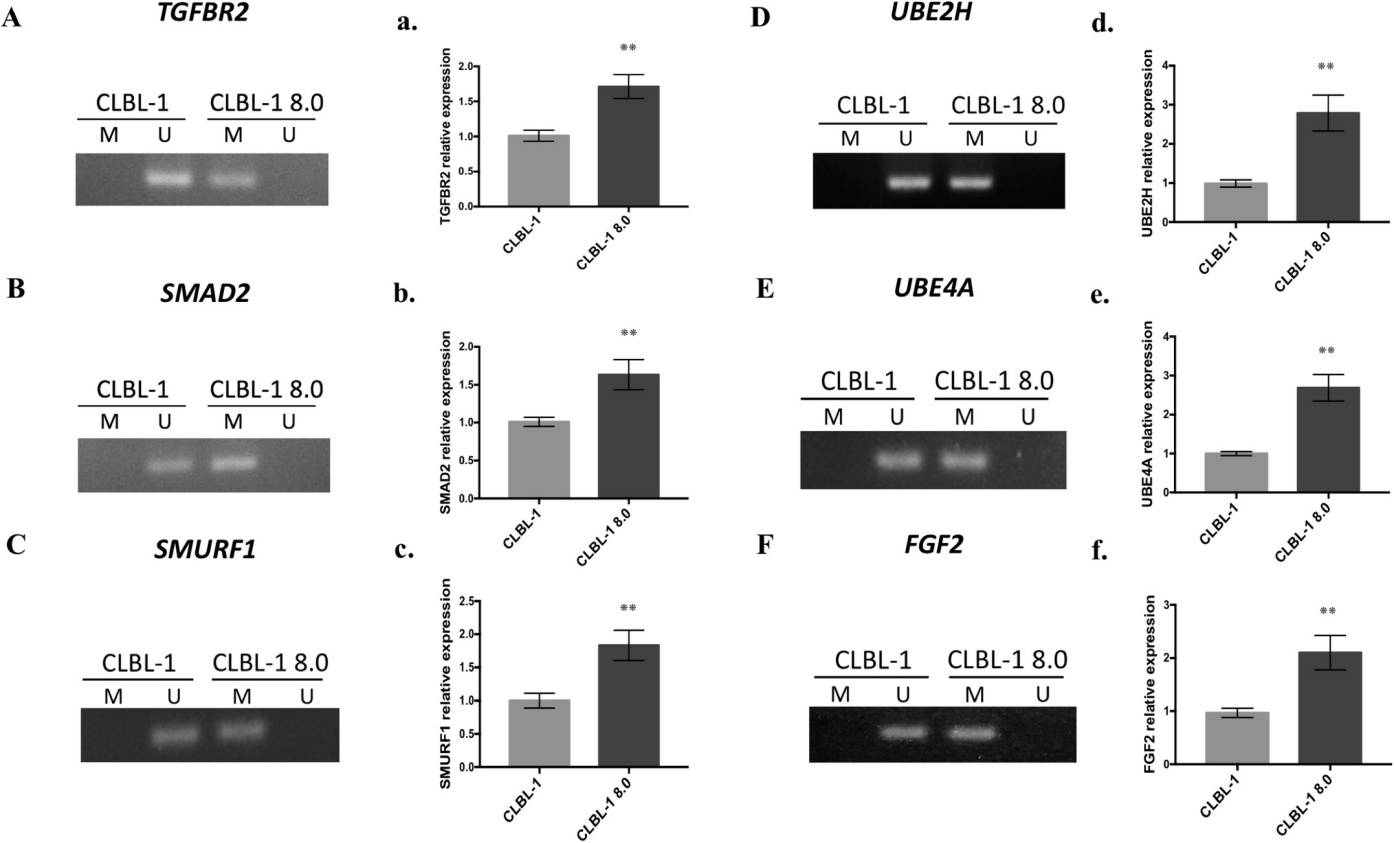
DNA hypermethylation in gene-body regions of *TGFBR2*, *SMAD2*, *SMURF1*, *UBE2H*, *UBE4A*, *and FGF2* increased their mRNA expression levels in CLBL-1 8.0. (A-F) DNA methylation of these genes analyzed by MSP. Primer pairs were designed to amplify methylated (M) or unmethylated (U) genomes. (a~f) The mRNA expressions of these genes in CLBL-1 and CLBL-1 8.0 cells. *GAPDH* and *OAZ-1* were used as internal controls and showed similar results. Data standardized by *GAPDH* alone were presented. Error bars represent the SEM of the results from 3 independent experiments. ** P *< 0*.*01*.

Furthermore, the methylation status within the gene-body regions of TGFBR2, FGF2, as well as the hub genes, were validated by MSP to reveal their specific CpG islands were hypermethylated (Fig 4A–4F). Concomitantly, the mRNA expression of these genes was confirmed and increased significantly in CLBL-1 8.0 cells (Fig 4a–4f).

### Suppressing TGF-β signaling pathway reduced doxorubicin-resistance of CLBL-1 8.0

**Fig 5:**
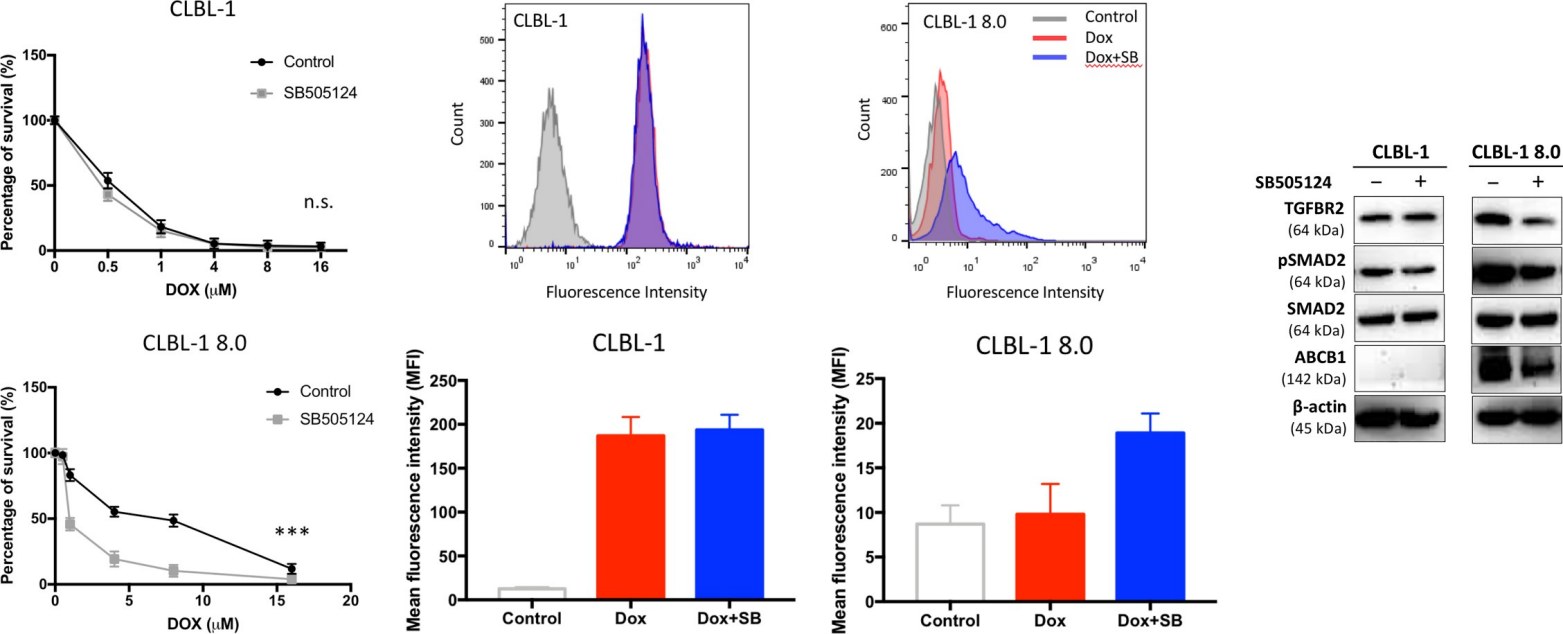
Inhibiting TGF-β signaling pathway reduced Dox-resistance of CLBL-1 8.0. (A, B) Cytotoxicity assay in CLBL-1 and CLBL-1 8.0 cells with the treatment of TGF-β receptor inhibitor, SB505124 (5 μM), and doxorubicin (Dox). Cytotoxicity assay was performed in triplicate, and error bars represent the SEM of the means. n.s., p > 0.05; ***, p ≤ 0.001; Dox: doxorubicin; Control: DMSO vehicle. (C) Representative dot plots showing fluorescence channel analysis and (D) quantitative comparison of Dox accumulation in CLBL-1and CLBL-1 8.0 cells after incubation with free Dox or SB505124 (5 μM) for 2 h at 37°C. Control: no doxorubicin treatment. (E) Suppressing TGF-β/Smad signaling axis by SB505124 (5 μM) for 24 hr reduced the quantities of ABCB1/P-gp expression in CLBL-1 8.0 cells.

The hub gene analysis and verification revealed that TGF-β signaling axis is significant in the development of drug resistance in CLBL-1 8.0. Several previous studies also reported that TGF-β induces chemo-resistance in a variety of cancers [24–27]. In order to measure the effect of TGF-β on doxorubicin-resistance of CLBL-1 and CLBL-1 8.0, we conducted *in vitro* cytotoxic assays following treatment with doxorubicin and a TGF-β inhibitor. Treating CLBL-1 cells with the TGF-β inhibitor, SB505124 (5 μM), did not alter their sensitivity to doxorubicin; however, it significantly reduced the cell viability of dox-treated CLBL-1 8.0 compared to the control treatment (p ≤ 0.001) (Fig 5A & 5B). Furthermore, doxorubicin accumulation in SB505124-treated CLBL-1 8.0 cells was also significantly increased compared to vehicle control (p ≤ 0.01) (Fig 5C & 5D).

TGF-β/Smad 2 signaling transduction was found to be upregulated in canine lymphoma cell lines to increase the mRNA quantities of *ABCB1* (ATP Binding Cassette Subfamily B Member 1, also known as P-glycoprotein, P-gp), the gene encodes efflux pump [28]. Our previous study also showed ABCB1/P-gp is overexpressed in CLBL-1 8.0 to mediate the doxorubicin resistance [13]. When Western blotting for the TGF-β/Smad pathway and ABCB-1 was performed, the quantities of phospho-Smad2 and ABCB1 of the CLBL-1 8.0 treated by SB505124 were found to be significantly decreased (Fig 5E). Taken together, these results were in accordance with our MethylCap-seq and RNA-seq findings to indicate the role of TGF-β signal pathway in developing the dox-resistance of CLBL-1 8.0.

## Discussion

Doxorubicin resistance is a major challenge in the treatment of cDLBCL. The mechanism of drug resistance is complicated, since both genetic mutation and aberrant DNA methylation play critical roles. Hence, it is particularly challenging to identify potential essential genes and pathways associated with drug resistance. In this study, we primarily focused on differential gene expression regulated by aberrant DNA methylation. To conduct a comprehensive analysis, MethylCap-seq and RNA-seq were carried out, and the sequencing results were analyzed integratively.

Previous studies have shown that gene body methylation, which is positively correlated with gene expression, plays a role in cancer [7–9]. We performed that analysis on hypermethylated/upregulated genes in gene-body regions to identify enriched GO terms and KEGG pathways. Most of these KEGG pathways were related to cancer, as expected. The “TGF-β signaling pathway” was identified as an enriched KEGG pathway in gene-body regions. TGF-β signaling was significantly increased in HCT116 human colon cancer cells treated with doxorubicin, and inhibition of TGF-β signaling markedly increased the sensitivity of HCT116 cells to doxorubicin [24], suggesting the role of elevated TGF-β signaling in resistance to doxorubicin. Also, it has been showed that TGF-β signaling conducted the increased gene expression of drug efflux pumps *ABCB1* and *LRP* in the side populations of canine lymphoma cell lines [28]. The enriched TGF-β signaling pathway identified in our study suggests that this pathway may contribute to doxorubicin resistance in cDLBCL. Besides, in the present study, several components of this pathway (i.e., *BMPR2*, *TGFBR2*, *ZFYVE16*, *CREBBP*, *SMURF1*, *SMAD2*, *SMURF2*) were found to be hypermethylated and upregulated in gene-body regions in CLBL-1 8.0. TGFBR2 (TGF-β Type II receptor) is a constitutively active serine/threonine/tyrosine kinase that forms a heterotetramer with Type I receptor upon TGF-β binding [26]. Elevated expression levels of TGFBR2 has been reported in a doxorubicin-resistant breast cancer MCF-7 cell line when compared to a sensitive MCF-7 cell line [27]. Increased expression levels of TGFBR2 might stimulate epithelial-mesenchymal transition (EMT) in multidrug-resistant MCF-7 cells, suggesting a relationship between EMT and drug resistance; however, this relationship is complicated and hence requires further research [27]. Our result showing upregulation of TGFBR2 in doxorubicin-resistant cDLBCL cells is in line with this study. When TGFBR2-mediated TGF-β signaling was inhibited by SB505124, the decreased cell viability and the amounts of phospho-Smad2 and ABCB1/P-gp, as well as the increased doxorubicin accumulation in CLBL-1 8.0 cells have proven the result in our epigenomic and transcriptomic findings that the TGFBR2-mediated TGF-β signal transduction plays a significant role in doxorubicin resistance in cDLBCL.

Hypermethylated/upregulated genes in gene-body regions belonging to the enriched KEGG pathway (p < 0.05) were further mapped to the PPI using the STRING database. Among this PPI network, 5 hub genes, defined as genes with the top 10% of connectivity in candidate modules [23], were identified, including *SMURF1*, *UBE4A*, *UBE2H*, *SMAD2* and *FBXL15* (homologous to human SMURF2), with *SMURF1* being the most essential one. Of note, 4 of these genes, namely, *SMURF1*, *UBE4A*, *UBE2H* and *FBXL15*, were involved in “ubiquitin-mediated proteolysis”. Ubiquitin-proteasome complex, which controls the degradation of intracellular proteins, is one of the two major protein degradation systems in the cell, while the lysosomal pathway, as the other primary protein degradation system, degrades extracellular proteins imported into cells [29, 30]. It has been reported that proteasome plays a crucial role in cancer cell proliferation, drug resistance development and inhibition of chemotherapy-induced apoptosis [31], and that the level of proteasome activity is elevated in cancer cells [29]. SMURF1 (Smad ubiquitin regulatory factor 1) is a critical E3 ubiquitin ligase targeting substrate proteins for ubiquitination and proteasomal degradation [32, 33]. Increasing evidence has shown that SMURF1 is a potential tumor-promoting factor in various cancers, including pancreatic cancer, breast cancer, colorectal cancer, clear cell renal cell carcinoma, head and neck squamous cell carcinoma, and gastric cancer [33–39]. Although no studies to date have indicated the role of SMURF1 in both human and canine DLBCL, the identification of *SMURF1* as one of the hub genes in our research reveals that this gene may be related to doxorubicin resistance in cDLBCL.

The study has potential limitations. Though whole methylome and RNA transcriptome were compared in CLBL-1 and doxorubicin-resistant CLBL-1 8.0 to reveal possible chemoresistant mechanisms, the verifications using clinical samples obtained from DLBCL patients with drug resistance should further prove the current findings and provide practical applications.

## Conclusions

The results of the current study provide a comprehensive analysis of the correlation between differentially expressed genes and aberrantly hypermethylated genes in promoter and gene-body regions, and they also reveal associated key pathways and specific genes involved in the development of doxorubicin resistance in cDLBCL.

## Supporting information

S1 File (DOCX)

S1 Raw images (PDF)
