# Supplementary material for: Genome-wide DNA methylation and RNA-seq analyses identify genes and pathways associated with doxorubicin resistance in a canine diffuse large B-cell lymphoma cell line
Source: PLoS One. 2021 May 7;16(5):e0250013. doi: 10.1371/journal.pone.0250013 (PMC8104391; doi:10.1371/journal.pone.0250013)
Supplement: S1 File — (DOCX) [file pone.0250013.s001.docx]

**S1 File**


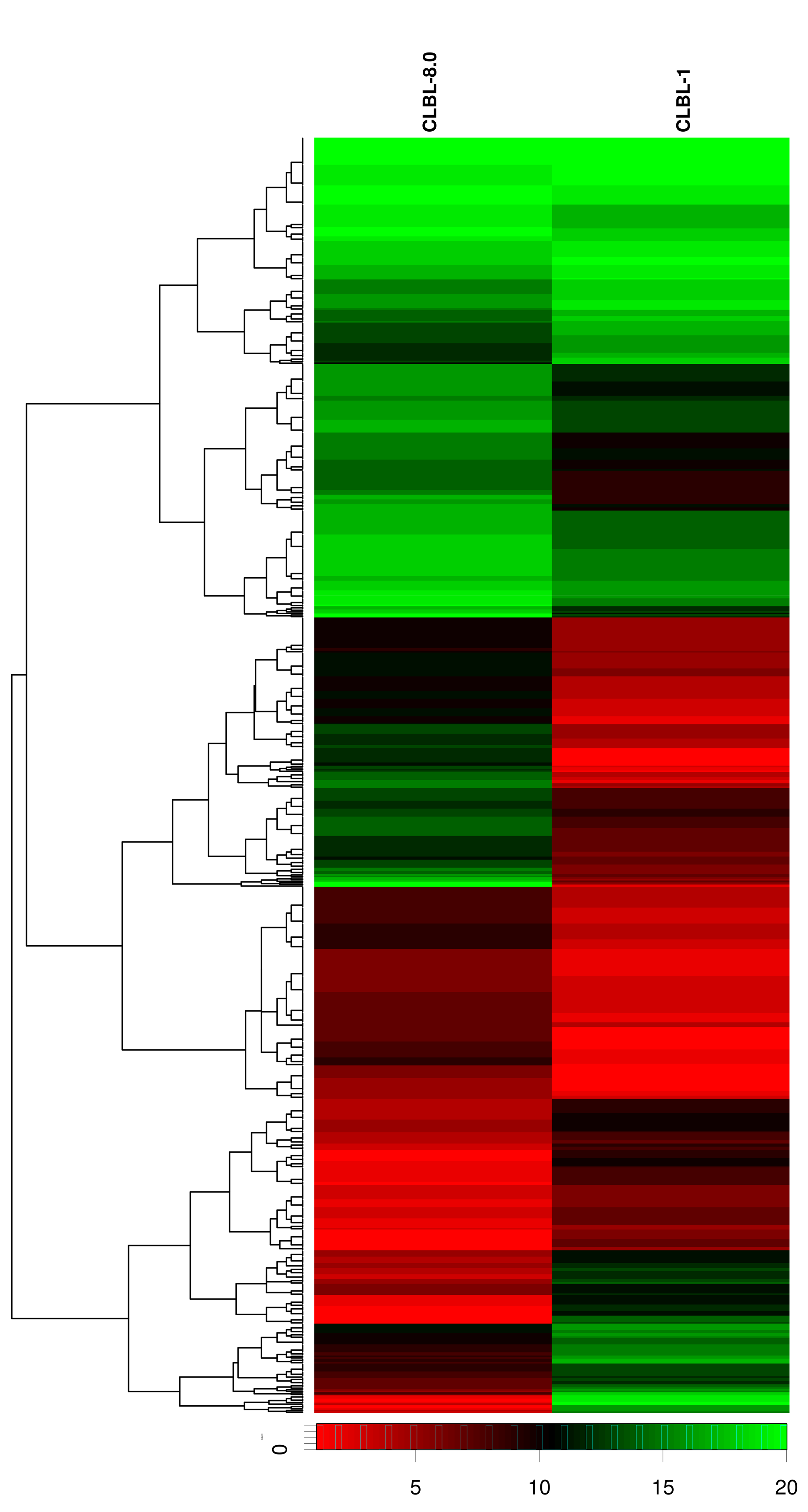


**S1 Fig**. Heatmap of RNA-Seq transcriptome analysis of CLBL-1 8.0 and CLBL-1 for the selected genes at a greater than log2-fold increase or decrease in expression level.

**S1 Table.** Primers for methylation-specific PCR

| **Primer set** |  | **Primer sequence (5'⟶3')** | **Position of the primers** | **NCBI Gene ID** |
| --- | --- | --- | --- | --- |
| TGFBR2-MSP-M | Forward | CGTTTTTGGGAATCGTTATAGTC | 13907519~13908283 | 477039 |
|  | Reverse | CCAAAATAATAACCAAATACTCGCT |  |  |
| TGFBR2-MSP-U | Forward | TGTTTTTGGGAATTGTTATAGTTGT |  |  |
|  | Reverse | CCAAAATAATAACCAAATACTCACT |  |  |
| SMAD2-MSP-M | Forward | GGGTTTGCGAGTGAGTTTTAC | 43720003~43720450 | 48044 |
|  | Reverse | TTCACAACAAACGAAATAAACGA |  |  |
| SMAD2-MSP-U | Forward | TTGGGTTTGTGAGTGAGTTTTAT |  |  |
|  | Reverse | TCACAACAAACAAAATAAACAAC |  |  |
| SMURF1-MSP-M | Forward | GGTTGTAGGTGGCGTTAAATC | 10452198~10452557 | 608821 |
|  | Reverse | ATACGATCATAATCTCAAACTCGTA |  |  |
| SMURF1-MSP-U | Forward | GGGTTGTAGGTGGTGTTAAATTG |  |  |
|  | Reverse | AAATACAATCATAATCTCAAACTCATA |  |  |
| UBE2H-MSP-M | Forward | TTAGGGATTTTTAGGTGGCGT | 658458~6958814 | 607816 |
|  | Reverse | AAAAAACAAACTCCATACACCGA |  |  |
| UBE2H-MSP-U | Forward | AGGTTTAGGGATTTTTAGGTGGT |  |  |
|  | Reverse | AAAAAAACAAACTCCATACACCAA |  |  |
| UBE4A-MSP-M | Forward | ATGTAGGGAATTTAACGTGG | 15348552~15348989 | 479418 |
|  | Reverse | AATAATTTTATATTAAAACAACCCGAA |  |  |
| UBE4A-MSP-U | Forward | TATGTAGGGAATTTAATGTGG |  |  |
|  | Reverse | AATAATTTTATATTAAAACAACCCAAA |  |  |
| FGF2-MSP-M | Forward | TATTTTAGGATTAGGGGGATCG | 17458436~17459047 | 403857 |
|  | Reverse | CTAAACCAAAAACAAACGCTA |  |  |
| FGF2-MSP-M | Forward | ATTTTAGGATTAGGGGGATT |  |  |
|  | Reverse | CTAAACCAAAAACAAACACTAAACTACTAC |  |  |

**S2 Table.** Primers for qRT-PCR

| **Gene** | **Gene ID** |  | **Sequence (5' --> 3')^a^** | **Amplicon size (bp)** | **Genbank accession number** |
| --- | --- | --- | --- | --- | --- |
| TGFBR2 | 477039 | Forward | GCCAACAACATCAACCACAACAC | 83 | XM_014106674 |
|  |  | Reverse | TAGACCTCAGCAAAGCGACCT |  |  |
| SMAD2 | 480144 | Forward | GTCATCTATCCTGCCGTTCACTCC | 113 | XM_005622832 |
|  |  | Reverse | TTTCTTCTTGTCCGTTCTGCTCTCC |  |  |
| SMURF1 | 608821 | Forward | GCCAACACAGACAACCTTCCA | 134 | XM_005621177 |
|  |  | Reverse | CACTCCACAGCAAACCCACAA |  |  |
| UBE2H | 607816 | Forward | AGGCGGAGTATGGAAAGTTAGAGTG | 125 | XM_022427233 |
|  |  | Reverse | GGAATCTGAGGAATGGGTCAGGG |  |  |
| UBE4A | 479418 | Forward | AAGAAATGGCAGTAGACCTGGAAGA | 88 | XM_844988 |
|  |  | Reverse | TCCTGAAGTAATAAGCGAGCGAAGA |  |  |
| FGF2 | 403857 | Forward | AATGTGTTACTGACGAGTGCTTCTT | 88 | XM_003432481 |
|  |  | Reverse | TACCAACTGGAGTATTTCCTTGACC |  |  |
| OAZ-1 | 476749 | Forward | CTGCTGTAGTAACCTGGGTC | 123 | NM_001127234.1 |
|  |  | Reverse | ACATTCAGCCGATTATCAGAGTA |  |  |
| GAPDH | 403755 | Forward | TGTCCCCACCCCCAATGTATC | 100 | NM_001003142.2 |
|  |  | Reverse | CTCCGATGCCTGCTTCACTACCTT |  |  |

1. The primer sequences of OAZ-1 and GAPDH were chosen on the basis of the published study (Mair et al., 2017, [18]). The primers for target genes were designed by Primer 3 (v.0.4.0).

**S3 Table.** GO terms for upregulated differentially expressed genes in CLBL-1 8.0 compared to CLBL-1

| Category | ID | Term | # of involve genes | P value | FDR | Involve gene list (gene name) |
| --- | --- | --- | --- | --- | --- | --- |
| GO_BP | GO:0006886 | intracellular protein transport | 19 | 6.73E-03 | 3.48E-02 | STAM,STX11,EVI5,SNX17,SYTL3,SYTL5,TBC1D10B,AP4M1,GGA1,LOC480654,SNX33,GGA2,COG7,TBC1D14,AP1S1,VPS16,STX2,STX6,AP4E1 |
|  | GO:0042771 | intrinsic apoptotic signaling pathway in response to DNA damage by p53 class mediator | 6 | 1.02E-02 | 3.51E-02 | HIPK1,USP28,CDKN1A,PMAIP1,TP63,CDIP1 |
|  | GO:0000209 | protein polyubiquitination | 11 | 1.05E-02 | 3.26E-02 | RNF41,RNF144A,RNF111,SHPRH,UBE4A,MARCH8,RNF125,SMURF1,RNF24,RUSC1,FBXO4 |
|  | GO:0030579 | ubiquitin-dependent SMAD protein catabolic process | 3 | 2.66E-02 | 4.58E-02 | RNF111,STUB1,SMURF1 |
|  | GO:2000145 | regulation of cell motility | 3 | 2.66E-02 | 4.34E-02 | FES,PKN2,CD81 |
|  | GO:0071404 | cellular response to low-density lipoprotein particle stimulus | 3 | 3.85E-02 | 4.97E-02 | NPC1,FCER1G,CD81 |
|  | GO:0033173 | calcineurin-NFAT signaling cascade | 3 | 3.85E-02 | 4.77E-02 | NFATC1,NFATC4,RCAN1 |
|  | GO:1902018 | negative regulation of cilium assembly | 3 | 3.85E-02 | 4.59E-02 | CEP97,CCP110,TCHP |
|  | GO:0001701 | in utero embryonic development | 15 | 4.37E-02 | 4.84E-02 | SMAD2,PLOD3,TANC2,MAPK8IP3,WDTC1,HINFP,ELL,PCGF2,ZMIZ1,OCRL,APBA3,HHEX,MIB1,BCL2L1,TGFBR2 |
|  | GO:0090023 | positive regulation of neutrophil chemotaxis | 4 | 4.50E-02 | 4.81E-02 | C3AR1,RAC1,LOC490356,CAMK1D |
| GO_CC | GO:0005794 | Golgi apparatus | 45 | 3.67E-04 | 1.43E-01 | TSC2,WDR44,STX11,USP33,LDLR,NPC1,ARV1,CDIPT,CAPN2,ZDHHC8,MYO6,RNF144A,GAS8,BHLHE40,CUBN,HEPACAM2,BACE1,PRKCE,GOLM1,OLFM3,CLN3,CLCN3,EVI5,GOLIM4,KIFAP3,STRN3,SNX17,MAPKAP1,EXT1,MGAT5,HIP1,CCDC91,GCC1,PLA2G4A,SRGN,YES1,CD44,PXYLP1,LMF1,DRAM2,GORAB,SLC30A7,ATP8B2,AP1S1,RUSC1 |
|  | GO:0005829 | cytosol | 72 | 1.14E-03 | 1.77E-02 | CAPN1,GLI1,PDXP,ICA1,NFATC4,PSTPIP2,CASTOR1,ABL1,WDTC1,CAPN2,AP4M1,RAP2A,CPNE3,AHCYL1,PDCD4,RAB29,DENND5A,SORT1,PGPEP1,ABHD10,SNX17,SERF2,DUSP3,AGTPBP1,DUSP7,ABHD14B,SH3RF1,SNX33,ARL6,ATP6V1A,CYTH3,NBEA,AP1S1,DVL1,BCL2L1,STX6,ELAC1,DNAJB4,CDKN1A,SRI,PLCD1,RANBP3L,TTC7B,RASAL1,FAM129B,PRMT6,RGS1,TBC1D14,GIMAP4,ATP6V1G2,WASL,AGBL5,PMAIP1,PRKCE,RAPGEF2,EVI5,DGUOK,HYAL2,LOC611915,SHMT1,TRIM8,TP63,TBC1D10B,RALGDS,PLA2G4A,YES1,LOC102154748,STXBP3,NFKBIA,DIXDC1,RAC1,IST1 |
|  | GO:0005764 | lysosome | 16 | 2.11E-03 | 2.18E-02 | CAPN1,TSC2,CTSF,CLN3,LDLR,CTSS,GUSB,VMA21,HYAL2,CAPN2,IDUA,HYAL3,FYCO1,DRAM2,DLA-DQA1,MARCH8 |
|  | GO:0005765 | lysosomal membrane | 17 | 3.87E-03 | 3.00E-02 | CD63,CLN3,HGSNAT,NPC1,SPPL3,STARD3NL,LAMTOR2,TMEM175,TRIM23,ATP6V1A,TMEM79,SPNS1,TLR3,CUBN,VPS16,GNB2,CLCN7 |
|  | GO:0005622 | intracellular | 54 | 7.67E-03 | 3.40E-02 | ZNF568,ZNF354A,STK38L,ANXA5,SYTL5,CAPN2,TRIM44,RHOBTB1,PIK3CA,ADCY9,DEF8,SH2D1B,PLXNA3,ZNF584,STAM,TRIM46,LOC489911,RCAN2,NOXO1,ARHGEF3,RCAN1,ARF5,STX6,PKN3,LOC100684414,ZNF688,PLCD1,RANBP3L,RASAL1,ANO6,LOC480654,DOCK9,ZNF862,ZFP2,ARF3,PKN2,CAPN15,UBL3,RNF125,ZNF597,SPSB2,PRKCE,EVI5,PITPNA,ZNF184,AGAP2,IRAK1BP1,KRBA1,TBC1D10B,RALGDS,TGFBR3,HPCAL4,GTPBP2,RAC1 |
|  | GO:0005768 | endosome | 15 | 1.17E-02 | 3.02E-02 | CLCN3,NPC1,UNC93B1,GRAP2,STEAP4,SNX30,SNX33,TMEM175,DGKH,RAB11FIP2,CUBN,VPS16,MARCH8,SNX7,PACSIN3 |
|  | GO:0005929 | cilium | 9 | 1.45E-02 | 3.46E-02 | ARL6,IFT122,CLUAP1,IFT22,LCA5,BBS12,TULP3,IFT46,WDR35 |
|  | GO:0005925 | focal adhesion | 26 | 1.83E-02 | 4.05E-02 | CAPN1,TLN2,USP33,SLC9A3R2,ZYX,ANXA5,SORBS3,ALCAM,CD99L2,CD81,CAPN2,LPXN,REXO2,CPNE3,DAG1,PNMA1,AIF1L,GNB2,PIK3R2,HYOU1,YES1,CD44,TSPAN4,FES,RAC1,ARPC1B |
|  | GO:0005913 | cell-cell adherens junction | 6 | 1.93E-02 | 3.99E-02 | NECTIN1,ZYX,SMAD7,NECTIN3,PVR,PTPRM |
|  | GO:0097546 | ciliary base | 4 | 2.48E-02 | 4.81E-02 | IFT122,PRKACB,TULP3,IFT52 |
|  | GO:0005802 | trans-Golgi network | 12 | 2.52E-02 | 4.60E-02 | CLN3,GGA2,OCRL,GSAP,RAB29,NBEA,DENND5A,RAC1,SCAMP1,AP4M1,BACE1,FURIN |
|  | GO:0070938 | contractile ring | 3 | 2.68E-02 | 4.15E-02 | PDXP,ALKBH4,DAG1 |
|  | GO:0072372 | primary cilium | 5 | 3.44E-02 | 4.85E-02 | GLI1,RAB15,RILPL1,AHI1,TULP3 |
|  | GO:0005770 | late endosome | 9 | 4.87E-02 | 4.87E-02 | SLA2,CLN3,CLCN3,LDLR,FYCO1,MTM1,F2R,VPS16,RAPGEF2 |
| GO_MF | GO:0035091 | phosphatidylinositol binding | 11 | 4.95E-03 | 3.07E-02 | FES,TNFAIP8L3,MTM1,NOXO1,SNX17,SNX30,HIP1,TULP3,SNX7,WDR35,SNX33 |
|  | GO:0070300 | phosphatidic acid binding | 4 | 9.80E-03 | 3.80E-02 | ATP13A2,PLCD1,MAPKAP1,RAPGEF2 |
|  | GO:0046872 | metal ion binding | 63 | 1.15E-02 | 3.24E-02 | ZNF568,GLI1,ZNF354A,ZFYVE16,HDAC9,ZNF518A,ZBTB14,ATP13A2,NUDT13,NME1,HINFP,ZNF775,ZNF821,ZNF646,MAZ,ZNF174,NTHL1,FYCO1,PARN,DEF8,ZC3H6,ZNF584,ZNF436,MZF1,ZBTB10,LOC489911,ZNF395,PDE4D,ANKZF1,NUBP2,FGD6,ZBTB12,ZSCAN29,YPEL3,GLI4,TIMP1,ZNF768,ZNF12,ZNF394,LOC100684414,ZNF688,PAPOLG,PTS,ZNF202,ZFP2,ZNF22,ZNF597,TGFBR2,PRKCE,ZKSCAN2,SLC2A4RG,ZFYVE27,ZNF74,ZNF655,SNAI2,ZNF184,ZSCAN25,MBNL3,HELZ2,KLF11,ZNF785,ZNF853,ZBTB22 |
|  | GO:0000981 | RNA polymerase II transcription factor activity, sequence-specific DNA binding | 15 | 3.25E-02 | 4.80E-02 | ZKSCAN2,FOXJ2,ZNF518A,FOXK1,ETV6,RUNX1,ELK1,ZNF202,LOC607692,ZNF174,KLF11,JUND,ZSCAN29,DMTF1,FAM200A |
|  | GO:0061630 | ubiquitin protein ligase activity | 14 | 3.83E-02 | 5.16E-02 | RNF111,UBE2H,RNF216,PJA2,BFAR,CBL,RNF149,RNF41,RNF144A,MARCH8,RNF125,SMURF1,RNF24,FBXO4 |
|  | GO:0005096 | GTPase activator activity | 16 | 4.15E-02 | 4.76E-02 | SYNGAP1,TSC2,ARHGAP26,RASAL2,EVI5,ELMOD2,AGAP2,RASAL1,TBC1D10B,LOC480654,AXIN1,OCRL,RGS1,TBC1D14,RGS22,RAPGEF2 |
|  | GO:0017137 | Rab GTPase binding | 7 | 4.76E-02 | 4.92E-02 | GAS8,EVI5,TBC1D14,RAB11FIP2,TBC1D10B,LOC480654,RAB11FIP1 |

**S4 Table.** GO terms for downregulated differentially expressed genes in CLBL-1 8.0 compared to CLBL-1

| Category | ID | Term | # of involve genes | P value | FDR | Involve gene list (gene name) |
| --- | --- | --- | --- | --- | --- | --- |
| GO_BP | GO:0042167 | heme catabolic process | 3 | 2.83E-03 | 3.11E-02 | HMOX1,BLVRA,BLVRB |
|  | GO:0000281 | mitotic cytokinesis | 5 | 3.62E-03 | 3.19E-02 | RACGAP1,SNX9,SNX18,KIF20A,SPTBN1 |
|  | GO:0006412 | translation | 13 | 7.48E-03 | 4.11E-02 | RPS18,LOC100685611,MRPL22,SLC25A11,RPS14,SLC25A6,MRPL35,RPL3,RPL32,RPL10A,SLC25A35,RPL18,MRPL20 |
|  | GO:0051607 | defense response to virus | 8 | 8.59E-03 | 4.20E-02 | APOBEC3Z3,ISG20,BST2,PRF1,CXCL10,EXOSC5,NLRC5,OAS1 |
|  | GO:0036089 | cleavage furrow formation | 3 | 9.06E-03 | 3.99E-02 | SNX9,SNX18,AURKB |
|  | GO:0001771 | immunological synapse formation | 3 | 9.06E-03 | 3.62E-02 | PRF1,DOCK2,DOCK8 |
|  | GO:0051988 | regulation of attachment of spindle microtubules to kinetochore | 3 | 1.33E-02 | 2.66E-02 | RACGAP1,KNSTRN,NEK2 |
|  | GO:0030334 | regulation of cell migration | 6 | 1.49E-02 | 2.85E-02 | ABI3,RACGAP1,LOC100683370,GAB1,MYSM1,LOC102152706 |
|  | GO:0042273 | ribosomal large subunit biogenesis | 4 | 1.72E-02 | 3.15E-02 | SDAD1,RPL26,LOC608703,NOP16 |
|  | GO:0000070 | mitotic sister chromatid segregation | 4 | 1.72E-02 | 3.15E-02 | KNSTRN,NEK2,CENPA,MIS12 |
|  | GO:0097190 | apoptotic signaling pathway | 5 | 2.04E-02 | 3.21E-02 | TFPT,TM2D1,P2RX4,TNFRSF14,ANXA6 |
|  | GO:0045071 | negative regulation of viral genome replication | 4 | 2.96E-02 | 4.34E-02 | SLPI,ISG20,BST2,OAS1 |
|  | GO:0032467 | positive regulation of cytokinesis | 4 | 2.96E-02 | 4.34E-02 | RACGAP1,AURKB,CENPV,CIT |
|  | GO:0051382 | kinetochore assembly | 3 | 3.00E-02 | 4.00E-02 | CENPA,CENPS,MIS12 |
|  | GO:0043928 | exonucleolytic nuclear-transcribed mRNA catabolic process involved in deadenylation-dependent decay | 3 | 3.00E-02 | 4.00E-02 | EXOSC8,CNOT8,EXOSC5 |
|  | GO:0030819 | positive regulation of cAMP biosynthetic process | 4 | 3.33E-02 | 4.31E-02 | AKAP5,ADORA2B,ADCY7,AKAP12 |
|  | GO:0006979 | response to oxidative stress | 6 | 3.60E-02 | 4.53E-02 | RRM2B,HMOX1,OXSR1,GAB1,NQO1,APOE |
|  | GO:0034501 | protein localization to kinetochore | 3 | 3.68E-02 | 4.49E-02 | KNL1,AURKB,MIS12 |
|  | GO:0051591 | response to cAMP | 3 | 4.40E-02 | 4.96E-02 | NDUFS4,PER1,STAT4 |
|  | GO:0006886 | intracellular protein transport | 11 | 4.52E-02 | 4.85E-02 | USO1,SNX9,ANKRD50,SNX18,STX8,SEC23A,TOM1L2,TBC1D10C,SYTL1,TBC1D8,TBC1D22B |
|  | GO:0007059 | chromosome segregation | 5 | 4.70E-02 | 4.70E-02 | NDEL1,KNSTRN,NEK2,SPC25,BIRC5 |
| GO_CC | GO:0016592 | mediator complex | 6 | 2.38E-03 | 1.05E-01 | MED31,PPARGC1B,MED9,CCNC,MED10,MED11 |
|  | GO:0000776 | kinetochore | 7 | 6.12E-03 | 4.49E-02 | NDEL1,KNSTRN,NEK2,CENPA,CENPS,AURKB,CENPV |
|  | GO:0022625 | cytosolic large ribosomal subunit | 7 | 1.10E-02 | 4.03E-02 | RPL32,RPL3,LOC100685611,RPL26,RPL10A,LOC608703,RPL18 |
|  | GO:0070062 | extracellular exosome | 83 | 1.17E-02 | 3.43E-02 | SLPI,LAMC1,SNX9,OXSR1,STOM,SYTL1,EPCAM,NQO1,P2RX4,DOCK2,ATP6V1B2,TPST2,TUBB4B,EIF4E,FSTL1,GRHPR,LCP1,CMTM6,SMIM1,PFAS,NUDT9,LOC100855532,MEST,SPTBN1,AKR1E2,PKD2,LOC488622,ACTA1,TNFSF10,SLC1A5,NDRG1,COTL1,SNX18,C9H17orf80,GM2A,HNRNPDL,COPS4,SCARB2,SYPL1,PGD,ANXA6,BLVRB,ANXA4,FERMT3,NAGA,B2M,CSK,RRM2B,HNRNPD,ERAP1,TUBB6,BST2,CD9,MPDU1,CD37,LOC608682,RAC2,USP14,GCA,ISOC1,CALM3,OLA1,NAMPT,SERBP1,PSMD14,SLC16A1,ABAT,RACGAP1,GPR155,RPS18,PTPRD,PFKL,KNL1,TRMT10A,HIST2H2AA4,PSMB6,COBLL1,STK26,TOM1L2,RARS,PMVK,APOE,BLVRA |
|  | GO:0051233 | spindle midzone | 4 | 1.21E-02 | 2.96E-02 | RACGAP1,AURKB,CENPV,UNC119 |
|  | GO:0005911 | cell-cell junction | 9 | 1.27E-02 | 2.79E-02 | SH3KBP1,CSK,ADD3,THEMIS,PKD2,LCP2,LIMD1,VAV1,SKAP1 |
|  | GO:0005654 | nucleoplasm | 53 | 1.89E-02 | 3.08E-02 | PSMD4,CENPV,PNN,TFPT,CCNDBP1,CGRRF1,PPIG,RANGRF,BMP2K,AURKB,PRKRA,ELAC2,RCHY1,FOSL2,SNX18,APTX,COPS3,HNRNPDL,COPS4,LUC7L3,NR4A2,DRG2,NUP35,BLVRB,HAT1,RAD51C,RRM2B,HNRNPD,ZNF148,ETHE1,ATF2,ADD3,LOC608682,BUB1,NAMPT,DCPS,SRA1,MIER1,RACGAP1,DNMT3B,TM2D1,LARP1,KNL1,HNRNPA3,STAT1,RBM22,NUP88,PSMB6,ETS2,CPT2,AURKAIP1,RARS,ITGB3BP |
|  | GO:0048471 | perinuclear region of cytoplasm | 18 | 2.34E-02 | 3.55E-02 | SEC31A,USO1,FZD5,CDKN3,RAD51C,STX8,SEC23A,STOM,P2RX4,STAT1,NDRG1,DAB1,EIF4E,STK26,HMOX1,ANXA6,AKAP6,ANXA4 |
|  | GO:0030529 | intracellular ribonucleoprotein complex | 5 | 3.67E-02 | 4.49E-02 | G3BP1,NUP62,HNRNPD,SSB,SRA1 |
|  | GO:0030496 | midbody | 7 | 3.68E-02 | 4.26E-02 | RACGAP1,KIF20A,NEK2,AURKB,BIRC5,ASPM,SEPT1 |
|  | GO:0000786 | nucleosome | 5 | 4.59E-02 | 4.81E-02 | HIST2H2AA4,LOC488289,HIST1H1T,CENPA,KAT6A |
|  | GO:0005730 | nucleolus | 25 | 4.65E-02 | 4.76E-02 | SDAD1,CRACR2A,USO1,RASL11A,TERT,RBPJ,NOP16,DAB1,TFPT,AGPS,GEMIN2,EXOSC5,SF3B3,SENP3,OLA1,RPL18,NOP53,ISG20,SPTBN1,CPTP,TRIM68,TRMT10A,STAT1,CPT2,APTX |
|  | GO:0030991 | mediator complex | 6 | 2.38E-03 | 1.05E-01 | MED31,PPARGC1B,MED9,CCNC,MED10,MED11 |
|  | GO:0016023 | kinetochore | 7 | 6.12E-03 | 3.85E-02 | NDEL1,KNSTRN,NEK2,CENPA,CENPS,AURKB,CENPV |
|  | GO:0005783 | cytosolic large ribosomal subunit | 7 | 1.10E-02 | 3.72E-02 | RPL32,RPL3,LOC100685611,RPL26,RPL10A,LOC608703,RPL18 |
|  | GO:0005913 | extracellular exosome | 83 | 1.17E-02 | 3.68E-02 | SLPI,LAMC1,SNX9,OXSR1,STOM,SYTL1,EPCAM,NQO1,P2RX4,DOCK2,ATP6V1B2,TPST2,TUBB4B,EIF4E,FSTL1,GRHPR,LCP1,CMTM6,SMIM1,PFAS,NUDT9,LOC100855532,MEST,SPTBN1,AKR1E2,PKD2,LOC488622,ACTA1,TNFSF10,SLC1A5,NDRG1,COTL1,SNX18,C9H17orf80,GM2A,HNRNPDL,COPS4,SCARB2,SYPL1,PGD,ANXA6,BLVRB,ANXA4,FERMT3,NAGA,B2M,CSK,RRM2B,HNRNPD,ERAP1,TUBB6,BST2,CD9,MPDU1,CD37,LOC608682,RAC2,USP14,GCA,ISOC1,CALM3,OLA1,NAMPT,SERBP1,PSMD14,SLC16A1,ABAT,RACGAP1,GPR155,RPS18,PTPRD,PFKL,KNL1,TRMT10A,HIST2H2AA4,PSMB6,COBLL1,STK26,TOM1L2,RARS,PMVK,APOE,BLVRA |
|  | GO:0016605 | spindle midzone | 4 | 1.21E-02 | 3.13E-02 | RACGAP1,AURKB,CENPV,UNC119 |
|  | GO:0005764 | cell-cell junction | 9 | 1.27E-02 | 2.94E-02 | SH3KBP1,CSK,ADD3,THEMIS,PKD2,LCP2,LIMD1,VAV1,SKAP1 |
|  | GO:0012505 | nucleoplasm | 53 | 1.89E-02 | 3.20E-02 | PSMD4,CENPV,PNN,TFPT,CCNDBP1,CGRRF1,PPIG,RANGRF,BMP2K,AURKB,PRKRA,ELAC2,RCHY1,FOSL2,SNX18,APTX,COPS3,HNRNPDL,COPS4,LUC7L3,NR4A2,DRG2,NUP35,BLVRB,HAT1,RAD51C,RRM2B,HNRNPD,ZNF148,ETHE1,ATF2,ADD3,LOC608682,BUB1,NAMPT,DCPS,SRA1,MIER1,RACGAP1,DNMT3B,TM2D1,LARP1,KNL1,HNRNPA3,STAT1,RBM22,NUP88,PSMB6,ETS2,CPT2,AURKAIP1,RARS,ITGB3BP |
| GO_MF | GO:0003735 | structural constituent of ribosome | 15 | 2.81E-03 | 4.12E-02 | RPS18,LOC100685611,RPL26,MRPL22,SLC25A11,RPS14,SLC25A6,MRPL35,RPL3,RPL32,RPL10A,LOC608703,SLC25A35,RPL18,MRPL20 |
|  | GO:0001104 | RNA polymerase II transcription cofactor activity | 5 | 1.18E-02 | 3.25E-02 | MED31,PPARGC1B,MED9,MED10,MED11 |
|  | GO:0003785 | actin monomer binding | 3 | 1.33E-02 | 2.79E-02 | LOC100683370,COBLL1,LOC102152706 |
|  | GO:0030676 | Rac guanyl-nucleotide exchange factor activity | 3 | 4.40E-02 | 4.96E-02 | DOCK2,VAV1,FARP2 |

**S5 Table.** KEGG pathways for upregulated differentially expressed genes in CLBL-1 8.0 compared to CLBL-1

| KEGG pathway | # of associated genes | P value | FDR | Involve gene list (gene name) |
| --- | --- | --- | --- | --- |
| Endocytosis | 31 | 1.83E-06 | 4.45E-04 | PSD, SMAP2, ZFYVE16, IL2RA, LDLR, SMURF2, RAB5B, CBL, RAB11FIP1, ARF3, WASL, RAB11FIP2, CAPZB, SH3GLB2, SMURF1, VPS37B, TGFBR2, VPS37A, STAM, SMAD2, KIF5C, ZFYVE27, GRK4, AGAP2, RNF41, CYTH3, HSP70, IL2RB, ARPC1B, IST1, ARF5 |
| Pathways in cancer | 36 | 6.17E-04 | 6.25E-02 | GLI1, CDKN1A, PTGER2, GNG2, ABL1, GNG3, CBL, RUNX1, CDKN2B, AXIN1, PIK3CA, CCNE2, RASSF5, ADCY9, GNG4, PRKACB, APC, GNB2, TGFBR2, PIK3R2, SMAD2, ELOB, RXRB, F2R, RALGDS, RB1, NFKBIA, MAPK8, MAX, RAC1, LPAR6, FGF2, TRAF3, DVL1, BCL2L1, CREBBP |
| Lysosome | 16 | 7.72E-04 | 6.25E-02 | CTSF, CD63, CLN3, HGSNAT, CTSS, NPC1, GUSB, IDS, AP4M1, GGA1, IDUA, LAMP3, GGA2, AP1S1, SORT1, AP4E1 |
| Glycosaminoglycan degradation | 6 | 2.67E-03 | 1.15E-01 | HYAL3, HGSNAT, GUSB, HYAL2, IDS, IDUA |
| HTLV-I infection | 24 | 2.83E-03 | 1.15E-01 | TLN2, SMAD2, IL2RA, NFATC1, IL1R1, CDKN1A, NFATC4, CDKN2B, ELK1, PIK3CA, SRF, ADCY9, RB1, NFKBIA, DLA-DQA1, HLA-DRB1, IL2RB, PRKACB, APC, DVL1, BCL2L1, TGFBR2, PIK3R2, CREBBP |
| Adherens junction | 11 | 2.85E-03 | 1.15E-01 | NECTIN1, YES1, SMAD2, CSNK2A1, NECTIN3, WASL, SNAI2, RAC1, TGFBR2, PTPRM, CREBBP |
| Ras signaling pathway | 21 | 7.04E-03 | 2.44E-01 | SYNGAP1, RASAL2, RIN1, RAB5B, GNG2, ABL1, GNG3, RASAL1, RALGDS, PLA2G4A, ELK1, PIK3CA, RASSF5, GNG4, MAPK8, RAC1, FGF2, PRKACB, GNB2, BCL2L1, PIK3R2 |
| Chronic myeloid leukemia | 10 | 1.00E-02 | 3.00E-01 | PIK3CA, CDKN1A, RB1, NFKBIA, ABL1, CBL, RUNX1, BCL2L1, TGFBR2, PIK3R2 |
| Colorectal cancer | 9 | 1.12E-02 | 3.00E-01 | AXIN1, SMAD2, PIK3CA, MAPK8, RAC1, APC, TGFBR2, RALGDS, PIK3R2 |
| RIG-I-like receptor signaling pathway | 9 | 1.24E-02 | 3.00E-01 | CASP10, TBKBP1, NFKBIA, MAPK8, OTUD5, IL12A, TRAF3, RNF125, MAVS |
| Pancreatic cancer | 9 | 1.48E-02 | 3.04E-01 | SMAD2, PIK3CA, RB1, MAPK8, RAC1, BCL2L1, TGFBR2, RALGDS, PIK3R2 |
| Wnt signaling pathway | 14 | 1.50E-02 | 3.04E-01 | FRAT1, NFATC1, CSNK2A1, NFATC4, CAMK2D, AXIN1, CXXC4, PORCN, MAPK8, RAC1, PRKACB, APC, DVL1, CREBBP |
| TGF-beta signaling pathway | 10 | 2.07E-02 | 3.86E-01 | ZFYVE16, SMAD2, SMURF2, SMAD7, LOC100855618, BMPR2, CDKN2B, SMURF1, TGFBR2, CREBBP |
| Small cell lung cancer | 10 | 2.39E-02 | 4.05E-01 | PIK3CA, RXRB, CCNE2, RB1, NFKBIA, MAX, TRAF3, CDKN2B, BCL2L1, PIK3R2 |
| Apoptosis | 8 | 2.62E-02 | 4.05E-01 | CAPN1, CASP10, PIK3CA, IL3RA, NFKBIA, CAPN2, BCL2L1, PIK3R2 |
| Hepatitis B | 14 | 2.74E-02 | 4.05E-01 | NFATC1, CDKN1A, NFATC4, MAVS, ELK1, CASP10, PIK3CA, CCNE2, RB1, TLR3, NFKBIA, MAPK8, PIK3R2, CREBBP |
| Hepatitis C | 13 | 2.83E-02 | 4.05E-01 | CLDN23, LOC488947, LDLR, CDKN1A, CD81, MAVS, PIK3CA, TLR3, NFKBIA, MAPK8, TRAF3, EIF2AK3, PIK3R2 |
| MAPK signaling pathway | 20 | 4.62E-02 | 6.23E-01 | MAPKAPK3, NFATC1, IL1R1, MAPK8IP3, DUSP3, DUSP6, DUSP7, MAPT, PLA2G4A, ELK1, SRF, HSP70, MAPK8, MAX, JUND, RAC1, FGF2, PRKACB, TGFBR2, RAPGEF2 |

S6 Table. KEGG pathways for downregulated differentially expressed genes in CLBL-1 8.0 compared to CLBL-1

| KEGG pathway | # of associated genes | P value | FDR | Involve gene list (gene name) |
| --- | --- | --- | --- | --- |
| Ribosome | 14 | 6.66E-04 | 1.08E-01 | RPS18, LOC100685611, RPL26, MRPL22, RPS14, MRPL35, RPL3, RPL32, RPL10A, LOC608703, RPL18, RPL34, MRPL20, MRPS18C |
| Fc epsilon RI signaling pathway | 9 | 9.13E-04 | 1.08E-01 | FCER1A, PIK3R3, RAC2, MAP2K3, PLA2G4B, PIK3CG, PIK3CD, LCP2, VAV1 |
| Natural killer cell mediated cytotoxicity | 9 | 1.29E-02 | 7.20E-01 | PIK3R3, PRF1, RAC2, PIK3CG, PIK3CD, LCP2, TNFSF10, VAV1, SH2D1A |
| Arginine and proline metabolism | 6 | 1.79E-02 | 7.20E-01 | CKMT1A, LOC477562, ALDH4A1, CARNS1, P4HA2, SRM |
| Progesterone-mediated oocyte maturation | 8 | 1.98E-02 | 7.20E-01 | CCNB1, PIK3R3, BUB1, PIK3CG, PIK3CD, ADCY7, ANAPC10, CCNB2 |
| Porphyrin and chlorophyll metabolism | 5 | 2.02E-02 | 7.20E-01 | FECH, HMOX1, HCCS, BLVRA, BLVRB |
| Epstein-Barr virus infection | 9 | 2.43E-02 | 7.20E-01 | PIK3R3, DLA88, JAK3, PSMD4, RBPJ, MAP2K3, PIK3CG, PIK3CD, PSMD14 |
| Regulation of lipolysis in adipocytes | 6 | 3.05E-02 | 7.20E-01 | PIK3R3, ADRB2, PIK3CG, PIK3CD, ADCY7, MGLL |
| RNA transport | 11 | 3.21E-02 | 7.20E-01 | POP4, PNN, GEMIN5, EIF4E, NUP88, GEMIN2, NUP62, ELAC2, NUP210, NUP35, EIF4B |
| mTOR signaling pathway | 6 | 3.96E-02 | 7.20E-01 | PIK3R3, EIF4E, ULK2, PIK3CG, PIK3CD, EIF4B |
| Sphingolipid signaling pathway | 9 | 3.97E-02 | 7.20E-01 | PPP2R2A, FCER1A, PIK3R3, DEGS1, CERS6, RAC2, PLD2, PIK3CG, PIK3CD |
| Chemokine signaling pathway | 11 | 4.42E-02 | 7.20E-01 | PIK3R3, JAK3, CXCL10, RAC2, DOCK2, PIK3CG, PIK3CD, ADCY7, GNGT2, VAV1, STAT1 |
| TNF signaling pathway | 8 | 4.65E-02 | 7.20E-01 | PIK3R3, CXCL10, ATF2, MAP2K3, CASP7, IFGGC1, PIK3CG, PIK3CD |
| Measles | 9 | 4.66E-02 | 7.20E-01 | PIK3R3, JAK3, PIK3CG, PIK3CD, TNFSF10, RCHY1, OAS1, STAT1, SH2D1A |
| Central carbon metabolism in cancer | 6 | 4.75E-02 | 7.20E-01 | PIK3R3, PDK1, PFKL, PIK3CG, PIK3CD, SLC1A5 |
