# Supplementary figures and images for: Genome-wide DNA methylation and RNA-seq analyses identify genes and pathways associated with doxorubicin resistance in a canine diffuse large B-cell lymphoma cell line
Source: PLoS One. 2021 May 7;16(5):e0250013. doi: 10.1371/journal.pone.0250013 (PMC8104391; doi:10.1371/journal.pone.0250013)

## S1\_raw\_images

**Fig. 4A~F**

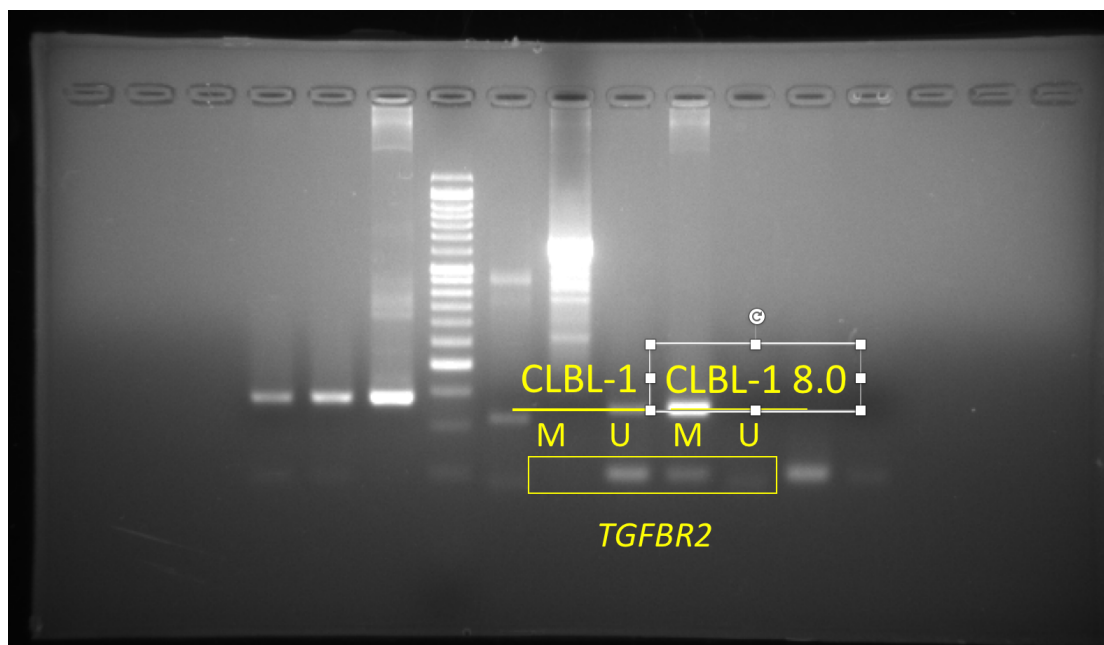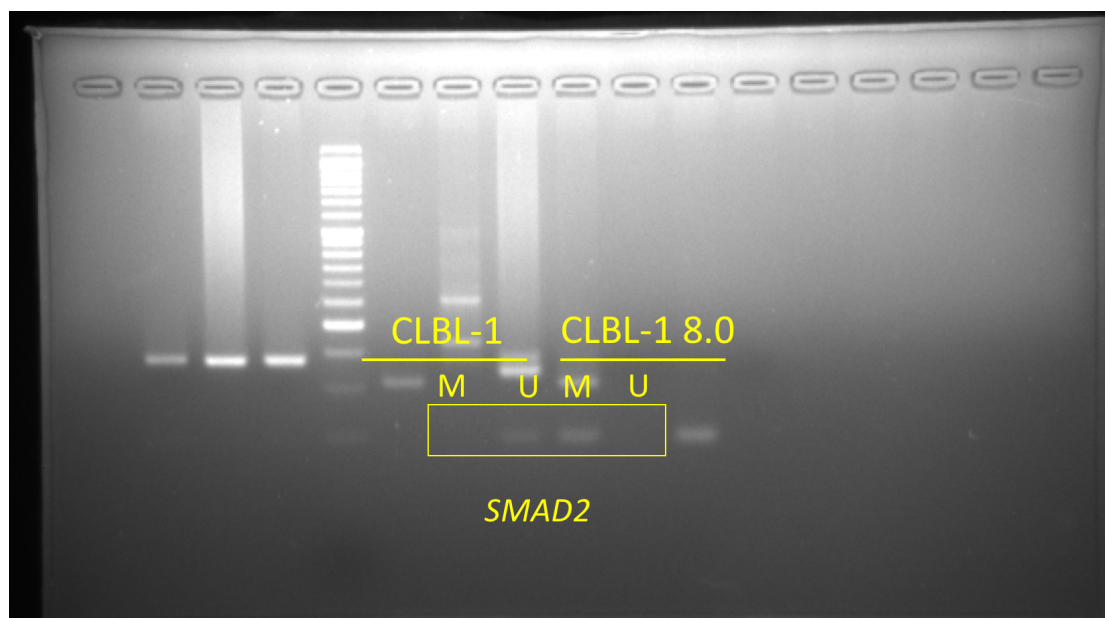

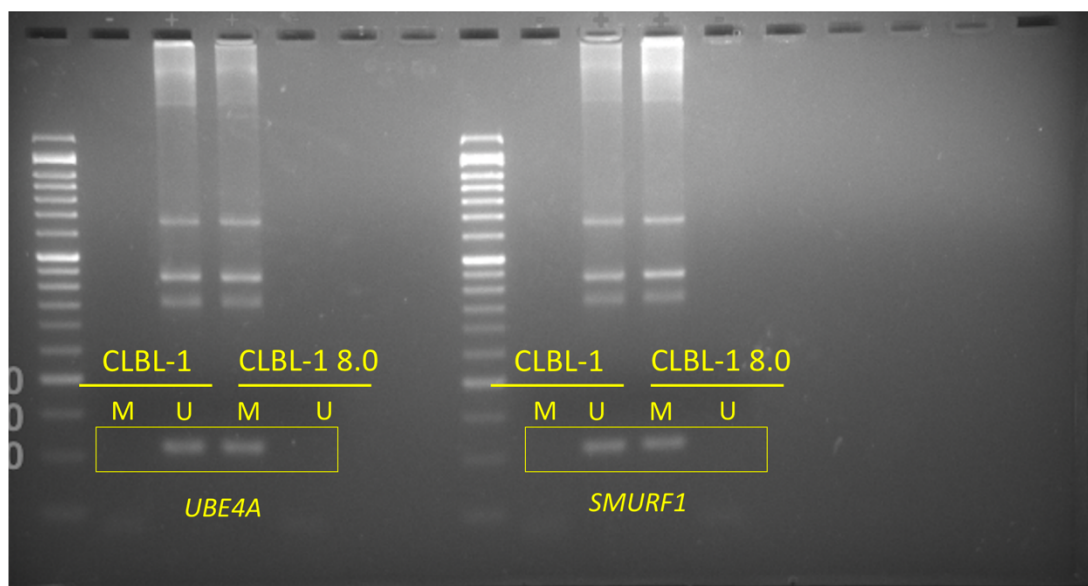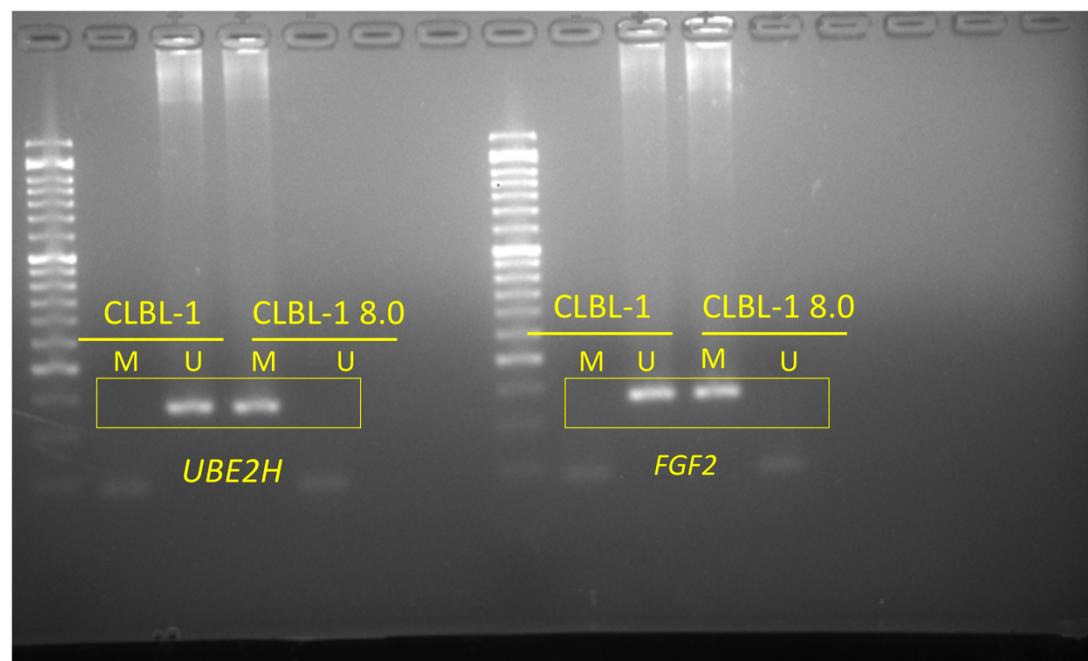

**Fig. 5E**

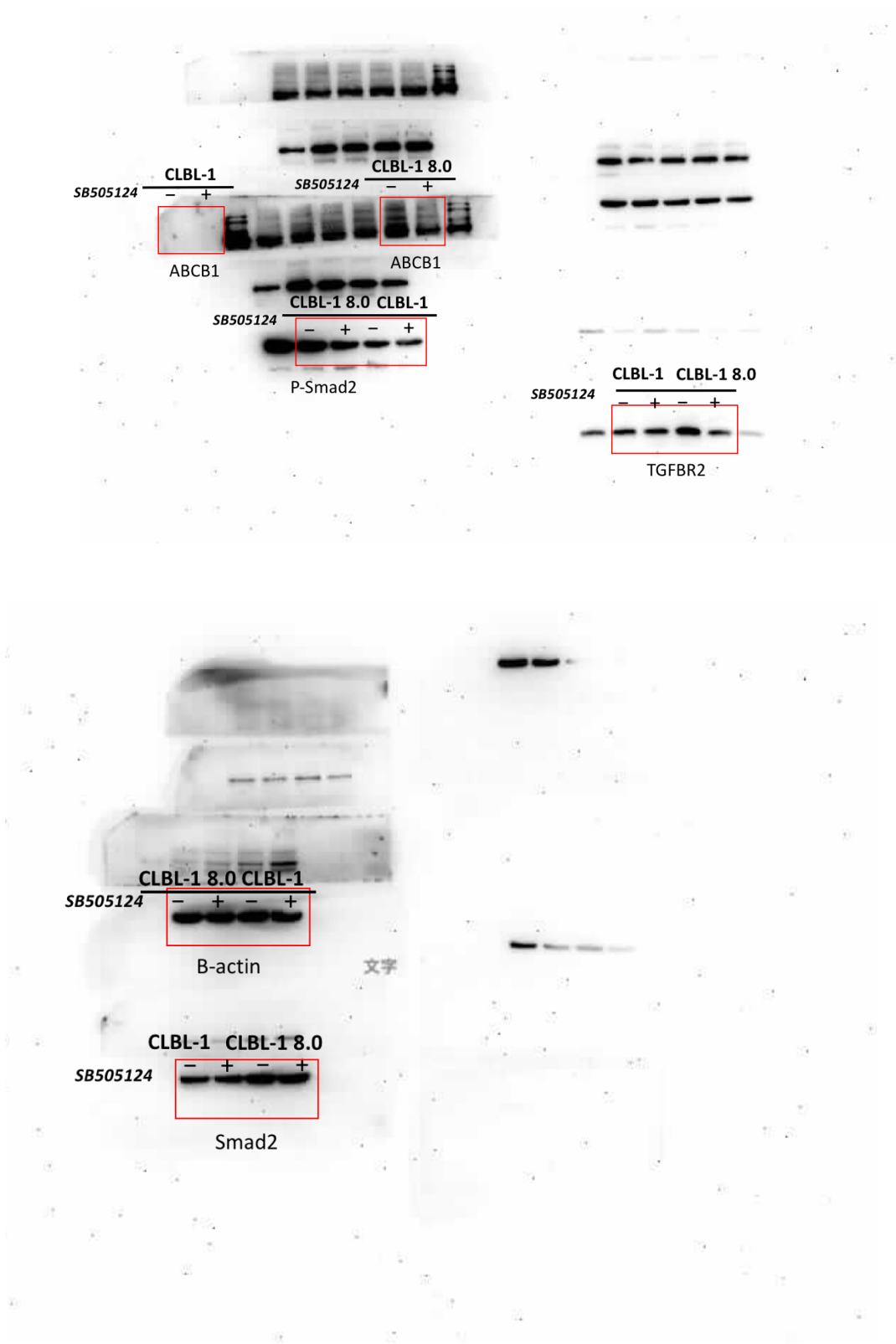

Supplement: S1 Raw images — (PDF) [file pone.0250013.s002.pdf]
